# Placenta Accreta Spectrum: A Review of Pathology, Molecular Biology, and Biomarkers

**DOI:** 10.1155/2018/1507674

**Published:** 2018-07-03

**Authors:** Helena C. Bartels, James D. Postle, Paul Downey, Donal J. Brennan

**Affiliations:** ^1^National Maternity Hospital, Holles Street, Dublin 2, Ireland; ^2^UCD School of Medicine, National Maternity Hospital, Holles Street, Dublin 2, Ireland

## Abstract

*Background*. Placenta accreta spectrum (PAS) is a condition of abnormal placental invasion encompassing placenta accreta, increta, and percreta and is a major cause of severe maternal morbidity and mortality. The diagnosis of a PAS is made on the basis of histopathologic examination and characterised by an absence of decidua and chorionic villi are seen to directly adjacent to myometrial fibres. The underlying molecular biology of PAS is a complex process that requires further research; for ease, we have divided these processes into angiogenesis, proliferation, and inflammation/invasion. A number of diagnostic serum biomarkers have been investigated in PAS, including human chorionic gonadotropin (HCG), pregnancy-associated plasma protein-A (PAPP-A), and alpha-fetoprotein (AFP). They have shown variable reliability and variability of measurement depending on gestational age at sampling. At present, a sensitive serum biomarker for invasive placentation remains elusive. In summary, there are a limited number of studies that have contributed to our understanding of the molecular biology of PAS, and additional biomarkers are needed to aid diagnosis and disease stratification.

## 1. Introduction

Placenta accreta was first described in 1937 by Irving et al. as failure of separation of the placenta from the uterine wall following delivery of the human fetus leading to the often used term morbid placental adherence [1]. The condition is characterised by invasive placentation which is associated with catastrophic haemorrhage. Varied terminology has been applied to this condition; however, recent guidelines suggested that placenta accreta spectrum (PAS), which includes accreta, increta, and percreta (defined below), be used going forward [2]. The condition is unique to human pregnancy with no animal correlate reported in the literature [3].

The incidence of PAS has increased substantially from 0.8 per 1000 deliveries in the 1980s to 3 per 1000 deliveries in the past decade, a phenomenon attributed to a rising global caesarean section rate [4]. PAS is associated with significant maternal morbidity and mortality, in particular, major obstetric haemorrhage and peripartum hysterectomy [5]. Mortality rates of up to 7% have been reported to be associated with PAS [6]. The most recent confidential inquiry into maternal mortality in the United Kingdom (MMBRACE-UK, 2017) highlighted the continued high maternal mortality associated with the condition [7].

The most important antenatal risk factor for PAS is the number of previous caesarean sections. In the presence of low-lying placenta (placenta previa) and three previous caesarean sections, a woman would have a 61% risk of PAS [8]. Antenatal diagnosis is a key element to improving maternal and perinatal outcome. Although dedicated ultrasound and MRI having improved antenatal diagnosis, between one half and two thirds of cases remain undiagnosed, resulting in poorer maternal outcomes [9, 10]. Hence, there is a continued need to develop and study methods to improve the antenatal diagnosis of PAS, particularly in the first trimester. This review aims to assess the existing evidence on the pathology, molecular biology, and biomarkers associated with PAS.

## 2. Pathology

PAS refers to a spectrum of abnormal placental adherence ranging from the subclinical (often microscopic) finding of adherent myometrial fibres within the basal plate to a dramatic presentation of placenta percreta, where there is placental invasion through the uterus and the serosa into the peritoneal cavity or bladder.

Traditionally, PAS is thought to occur as a consequence of a localised uterine injury (e.g., previous caesarean section) which can result in locally defective decidualisation/scarring and abnormal placental adherence in a subsequent pregnancy. Although typically attributed to previous caesarean delivery, even small disruptions to the lining of the uterus can result in subsequent placenta accreta [11]. While interactions between the maternal-fetal interface may also play a role in the pathogenesis of placenta accreta, this is beyond the scope of this article, and a number of studies describing the pathology of PAS in detail have been published [12–15].

Abnormal adherence of the placenta to the myometrium is established in very early pregnancy and can be subdivided into placenta accreta (where chorionic villi directly implant on to the myometrium), placenta increta (where chorionic villi invade into the myometrium), and placenta percreta (where chorionic villi invade through the myometrium and may involve surrounding structures). Placenta accreta is the most common component of PAS and accounts for 75% of cases. Mild forms of PAS may present as retained placenta that may require manual removal. When PAS is identified, the placentas are more often affected by chronic basal inflammation, changes of maternal vascular malperfusion, and retromembranous and subchorionic/intervillous haemorrhage [16].

**Figure 1:**
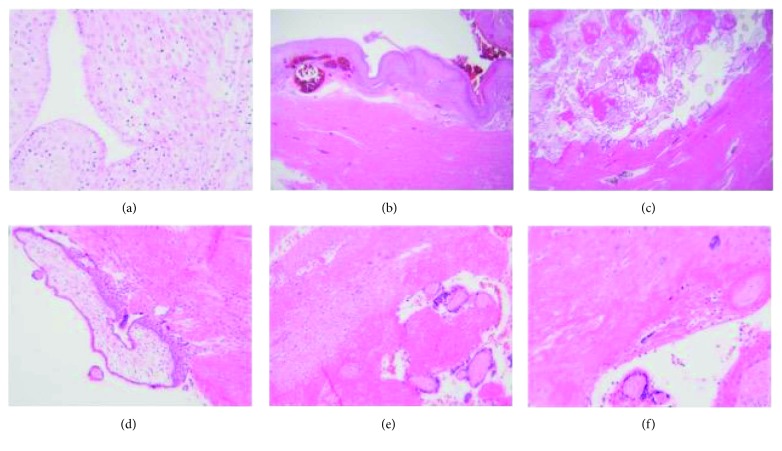
Histopathology of placenta accreta syndrome. (a) High-power picture of decidualised endometrium as a result of pregnancy. Stromal cells are large, pale, and polygonal. (b) Low-power image of decidualised endometrium on the surface with underlying congested myometrial blood vessels and myometrium. (c) Low-power image of PAS showing chorionic villi in direct contact with myometrium (no intervening decidua). (d) Chorionic villus with polar trophoblast invading myometrial muscle. (e) Nonadherent area of the same placenta where decidua is seen between villi (bottom right) and myometrium (top left). (f) PAS—chorionic villi in direct contact with muscle; a multinucleated extravillous trophoblast is seen in the top right.

The diagnosis of a PAS is made on the basis of histopathologic examination and characterised by an absence of decidua and chorionic villi are seen to directly adjacent to myometrial fibres (Figure 1). Although not visible macroscopically, microscopic examination of the placenta may confirm the presence of (placental) basal plate myometrial fibres (Figure 1); although this finding can be seen in normal pregnancies, their presence is thought to indicate abnormal placental separation. Perhaps more importantly, basal plate myometrial fibres are associated with an increased risk of a morbidly adherent placenta in a subsequent placenta/pregnancy [14].

## 3. Molecular Biology

**Figure 2:**
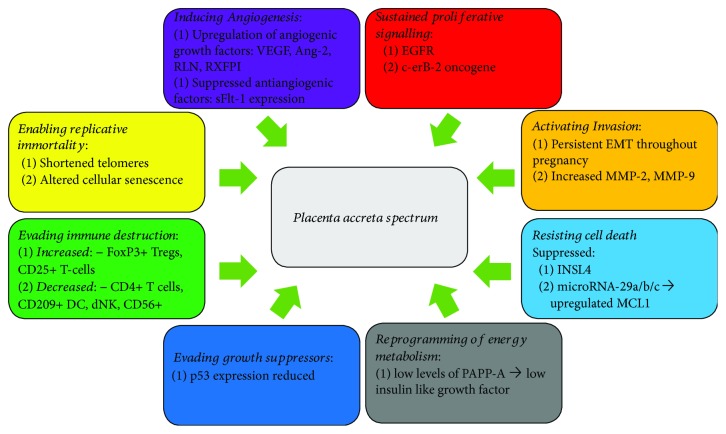
Similarities between PAS and cancer. Figure showing the 8 hallmarks of cancer as described by Weinberg and Hannahan and the similarities to the molecular biology of PAS [18].

The development of PAS is a complex multifactorial process. Normal placentae do not proceed beyond the inner third of the myometrium through tight spatial and temporal regulation; however, an invasive placenta proliferates and invades local structures in a similar fashion to a malignant tumour. The underlying molecular mechanisms of invasive placentation are poorly understood; proposed hypotheses include a combination of primary absence of the decidua or basal plate, abnormal maternal vascular remodelling, and excessive extravillous trophoblastic invasion [17]. Improved understanding of the molecular basis of other placental disorders such as preeclampsia suggests the inflammation and placental invasion may be closely related. A number of comparisons can be drawn between the microenvironment of PAS and tumour behaviour. Both conditions require an ability of cells to overcome the local immunological systems, activate invasion, and induce angiogenesis. In 2012, Hanahan and Weinberg outlined eight hallmark capabilities of tumours which allow them to invade and metastasise [18]. Herein, we use these eight hallmarks of cancer to highlight some of the molecular similarities between PAS and tumour development (Figure 2).

### 3.1. Inducing Angiogenesis

Extensive neovascularisation is clearly evident in the majority of PAS cases. Tseng and Chou demonstrated upregulation of a number of angiogenic growth factors, including vascular endothelial growth factor (VEGF) and angiopoietin-2 (Ang-2), in PAS lysates [19]. Reduced expression of antiangiogenic proteins such as VEGF receptor-2 (VEGFR-2), endothelial cell tyrosine kinase receptor Tie-2, and soluble fms-like tyrosine kinase 1 (sFlt-1) in syncytiotrophoblastic cells from PAS cases compared to normal placenta specimens suggests a proangiogenic phenotype [19].

Severe, early-onset preeclampsia is associated with inefficient physiological placental invasion and hypoperfusion, leading to increased sFlt-1 expression and ultimately the clinical phenotype of proteinuria and hypertension [20, 21]. In contrast, invasive placentation results in deep implantation and hyperperfusion, along with suppressed local sFlt-1 expression [22] as demonstrated by decreased expression of sFlt-1 in villous trophoblasts in PAS patients, specifically placenta increta and percreta [23].

PAS-related angiogenesis may not be restricted to the trophoblast. Placental relaxin (RLN) and its receptor (RXFPI) play an important role in angiogenesis in the endometrium by stimulating expression of VEGF [24]. Increased expression of RLN gene and protein has been demonstrated in the PAS basal plate, while the receptor RFXP1 is overexpressed in both the basal plate and villous trophoblast in PAS specimens compared to controls suggesting that PAS may produce a number of autocrine and paracrine factors that promote the upregulation of angiogenic-stimulating factors combined with a suppression in antiangiogenic factors leading to extensive neovascularisation [25].

### 3.2. Sustained Proliferative Signalling

Histological studies have demonstrated that the proliferative index and apoptotic rates of implantation site intermediate trophoblastic cells do not differ between PAS and normally implanted placenta [26, 27]. However, these comparisons were made between PAS specimens and normal placentae. A more informative comparison would be to compare PAS specimens and those taken from placenta previa with no evidence of adherence taken from women with a scarred uterus. Placenta previa is a significant risk factor for placenta accreta, present in 50% of cases [2]; hence, women with placenta previa and a previous uterine scar are a more suitable control group to identify histological differences between these women, who are at high risk of PAS, and those who developed PAS. Likewise, differential expression of epidermal growth factor receptor (EGFR) and c-erbB-2 oncogene in syncytiotrophoblast of PAS specimens has been described; however, again, the controls used are questionable, as many were taken from unscarred uteri [28]. The differential expression of these proteins in the syncytiotrophoblast but not in the invasive extravillous trophoblast suggests the possibility of a proliferative microenvironment comparable to recent advances in oncology research and the possibility that the syncytiotrophoblast may generate autocrine and paracrine factors that help promote proliferation of the extravillous trophoblasts. In order to further delineate this theory, comparative studies in women with placenta previa and a previous uterine scar are needed.

### 3.3. Resisting Cell Death

Placental apoptosis is an important process in the development of normal placentation [29]. Insulin-like protein 4 (INSL4), produced by the placenta, plays an important role in the inhibition of excessive placental proliferation by inducing apoptosis [30]. Decreased INSL4 gene expression in both the invasive and the noninvasive areas of extravillous trophoblasts has been demonstrated in patients with PAS when compared to gestational age-matched controls suggesting that abnormal invasion is a more generalized process due to the failure of normal apoptosis [25].

These findings are supported by Yongzhong et al., who demonstrated that microRNA-29a/b/c (miR-29a/b/c) inhibits apoptosis of implantation site intermediate trophoblasts in PAS [31]. The authors found significantly reduced expression of miR-29a/b/a in PAS specimens and suggest this downregulation of miR-29a/b/c contributes to trophoblast cell survival by upregulating myeloid cell leukemia-1 (MCL1), an antiapoptotic protein known to play a role in cancer survival.

### 3.4. Evading Immune Destruction

Any successful pregnancy depends on a fetoplacental unit-mediated suppression of the host immune response to prevent maternal rejection. As previously discussed, PAS is often associated with a chronic basal inflammation. The significance of leukocyte subpopulations and their contribution to excessive invasion of extravillous trophoblasts (EVT) in PAS requires further investigation; however, initial studies are intriguing. Ernst et al. demonstrated an increased lymphocytic infiltrate at the implantation site in a series of 101 PAS specimens compared to patients with placentas sent for examination due to a history of maternal malignancy with no clinical suspicion of PAS [32].

Immunohistochemical assessment of PAS specimens demonstrated significantly fewer CD4+ T-cells but a significant increase in FoxP3+ Tregs cells and also a slight increase in CD25+ T-cells compared to normal pregnancies all suggestive of a suppressive T-cell response [33]. PAS cases were also associated with significantly fewer immature nonactivated CD209+ dendritic cells. The Treg concentration correlated with increased EVT invasion and with differences in the density of dendritic cells suggests that increased invasion of the trophoblasts may be due to immunological dysfunction of decidua characterised by a suppressed T-cell response.

Decidual natural killer cells (dNK) are a unique subset of natural killer cells that play a critical role in the early phases of pregnancy. dNK cells secrete a variety of cytokines and angiogenic factors that are critical for a successful pregnancy and play a particularly important role in trophoblast invasion by establishing fetal tolerance [33]. The dNK cell population is significantly lower in PAS specimen, suggesting that dNK cell density inversely correlates with the degree of EVT invasion [34].

### 3.5. Activating Invasion

EVT cells in normal intrauterine pregnancy show a clear invasive front, whereas those affected by PAS were found to lack a defined invasion front with an irregular myometrial interface. This may be due to a number of processes.

Epithelial-to-mesenchymal transition (EMT) is a developmental program that results in the conversion of immotile epithelial cells into migratory mesenchymal cells [36]. While EMT is important to ensure the normal invasion and attachment of the placenta to the myometrium in the first trimester, EMT invasion should not persist throughout pregnancy. An abnormally aggressive EMT that continues throughout pregnancy has been demonstrated to play an important role in the development of PAS [37]. Duzyj et al. examined histological samples from 23 patients with PAS compared to a control group of 25 patients with a normal pregnancy and 21 with nonadherent placenta previa [37]. EVTs continued to display EMT features on third trimester hysterectomy specimens in the PAS group, demonstrated by coexpression of cytokeratin-7 and vimentin, when EMT should have ceased. These data suggest persistent EMT throughout pregnancy may be an important factor in the migratory behaviour of EVTs in PAS.

Matrix metalloproteinase (MMP) is a group of enzymes that play an important role in the penetration of trophoblast cells by degrading the extracellular matrix [38]. Specifically, the gelatinases MMP-2 and MMP-9 are found in high concentrations in the placenta [39]. There is conflicting evidence about their significance in PAS. Tseng et al. found no significant upregulation of MMPs in PAS specimens. However, Kocarslan et al. demonstrated stronger expression of MMP-2 in PAS specimens in a case-control study including 25 patients with PAS [39]. A further study found increased upregulation of MMP-9 and MMP-2 in PAS specimens compared to normal placentae [40].

Chen et al. investigated the role of MARVELD1, a nuclear protein that inhibits cell migration in mice [41, 42]. Knockout of placental MARVELD1 in a transgenic mouse model induced the downregulation of integrin *β*4 which resulted in increased trophoblast cell invasion. Interestingly, MARVELD1 is highly expressed in many different tissue types, downregulated in many cancers, and epigenetically silenced via DNA methylation, further demonstrating the similarities between cancer and PAS at the molecular level [43].

In summary, part of the ability of increased invasion of trophoblast cells in PAS appears to be explained by an overly aggressive and persistent EMT, increased activity of MMP enzymes, and changes in cellular adhesion proteins.

### 3.6. Enabling Replicative Immortality/Evasion of Growth Suppression

Cellular senescence is a permanent cell cycle arrest in response to DNA damage caused by a variety of factors such as oncogenes, oxidative stress, and telomere dysfunction [44]. Cellular senescence also occurs in the normal syncytiotrophoblast [45]. Tzadikevitch et al. compared known senescence-associated makers (p21, p15, p16, and the tumour suppressor protein p53) and telomere length in placenta percreta biopsies and normal placental gestational aged matched controls [45]. PAS specimens had shortened telomeres and altered senescence expression that was p21 dependent, compared to normal placentas which are controlled by p16 and p53. Although there is a paucity of data on tumour suppressor genes in PAS, the same paper demonstrated significantly lower expression of p53 in the PAS specimens compared to controls.

### 3.7. Reprogramming of Energy Metabolism

This is an area that requires further study. However, there may be some circumstantial evidence of a change in intracellular metabolism in PAS. Pregnancy-associated plasma protein A (PAPP-A) also known as Pappalysin-1 is a protein encoded by the PAPP-A gene in humans, located on human chromosome 9q33.1 [46]. First identified in 1974, PAPP-A is a novel zinc metalloproteinase synthesized by the syncytiotrophoblast and secreted into the maternal circulation in increasing concentrations until term [47, 48].

PAPP-A's main substrates are insulin-like growth factor-binding proteins [49]. PAPP-A increases the local bioavailability of insulin-like growth factor (IGF) by cleaving the inhibitors IGFBP-4 and -5 (insulin-like growth factor-binding protein-4 and -5); however, its function is not well understood) [50–53]. Low levels of PAPP-A are associated with increased levels of IGFBP protein and subsequently low levels of free IGF. IGF controls the uptake and transport of glucose and amino acids in trophoblasts and plays a role in autocrine and paracrine invasion of trophoblasts into the decidua [54]. The role of first trimester PAPP-A serum levels as a biomarker for PAS are discussed below; however, observations such as this suggest further work in the area of cellular energy metabolism in PAS may be important.

## 4. Biomarkers

For several years, investigators have attempted to identify maternal serum biomarkers that could be used to improve the accuracy of antenatal diagnosis of PAS. The use of ultrasound and MRI in the diagnosis of PAS has been extensively reviewed elsewhere [2, 55, 56]. Several placental and fetal hormones routinely used in the screening for aneuploidy have been found to be differentially expressed in the serum of women with PAS compared with those with placenta previa [57, 58]. More recently, there has been increasing interest in the role of cell-free fetal DNA (cffDNA) for screening and diagnosis of PAS.

### 4.1. Pregnancy-Associated Plasma Protein A (PAPP-A)

PAPP-A is a marker of placental syncitiotrophoblasts and reduced serum levels may serve as a marker for early placental dysfunction [59]. Low PAPP-A levels (</=0.4-0.5 multiples of the median (MoM)) have been associated with increased risk of developing preeclampsia, low birth weight, pregnancy loss, and preterm birth [60–67], all of which have been linked to abnormal trophoblastic invasion and placental development [68]. A number of studies have demonstrated that increased levels of first trimester PAPP-A are associated with PAS (Table 1).

Desai et al. published the first report of an association between first trimester PAPP-A and PAS [69], demonstrating that that first trimester PAPP-A levels were significantly increased in PAS cases compared to nonadherent placenta previa (Table 1). Although PAPP-A was elevated in PAS, it could not be used as a diagnostic marker because there was significant overlap with the distribution of unaffected pregnancies.

Thompson et al. subsequently examined whether differences existed in 516 routine first trimester maternal serum PAPPA-A measurements between normal pregnancies, placenta previa, and PAS and demonstrated that PAPP-A levels were significantly elevated in PAS cases with PAPPA-A showing a significantly different distribution from controls (*P* = 0.002). In a cohort of 236,714 singleton pregnancies, Lyell et al. identified 37 cases of PAS and 699 placenta previa controls [70]. Among multiparous women with placenta previa, first trimester PAPP-A values greater than 2.63 MoM conferred a nearly ninefold increased risk of PAS (95% confidence interval (CI) 2.8–27.4) independent of prior caesarean deliveries and a twenty-three- and thirty-six-fold increased risk for morbidly adherent placenta in the setting of one and two prior caesarean deliveries, respectively. Despite the large population dataset used in this study, an important limitation of this study was the diagnosis of morbidly adherent placenta was based on medical billing codes without surgical or pathologic confirmation.

### 4.2. Human Chorionic Gonadotropin (HCG)

hCG is a glycoprotein composed of 244 amino acids with a molecular mass of 36.7 kDa that is produced by the syncytiotrophoblast and maintains pregnancy by stimulating progesterone synthesis by the corpus luteum. A maximum level of approximately 100,000 iu/l is reached by 8–10 weeks of gestation and declines as placental steroid synthesis commences [71, 72]. hCG is a heterodimeric molecule composed of an alpha subunit that is identical to luteinising hormone, follicle-stimulating hormone, and thyroid-stimulating hormone and a beta (*β*) subunit that is unique. Proteolytic cleavage by trophoblast macrophages destabilises the molecule, thereby producing free *β*-hCG that is secreted into the maternal circulation [73, 74].

Whilst produced mainly by syncytiotrophoblasts, *β*-hCG is also synthesized by the fetal kidney and fetal liver. Besides maintaining the function of corpus luteum, free *β*-hCG also promotes angiogenesis, cytotrophoblast differentiation, and immunosuppression and blocks the phagocytosis of invading trophoblast cells, suggesting it should be considered as a marker of placentation and is used in multiparameter tests trying to predict placental function [75–77]. In the first trimester, reduced levels of free *β*-hCG (<0.5 MoM) have been associated with low birthweight and increased risk of spontaneous miscarriage. First trimester elevations in free *β*-hCG have not been associated with any adverse obstetric outcome. The converse holds true for the second trimester. Low levels of hCG have not been linked to adverse outcomes; however, elevated hCG (>2–4 MoM) has been associated with multiple complications such as large-for-gestational age placentae, retroplacental haematomas, and a low fetoplacental weight ratio [78]. Elevated hCG levels in the second trimester may be attributed to hypoxia-induced cytotrophoblastic proliferation which has been documented in histological studies. Decreased perfusion to the placenta may induce hypoxia, leading to cytotrophoblastic proliferation and subsequently to elevated levels of hCG [79].

Gestation-specific changes in hCG serum levels have been noted in cases of PAS. In 1999, Hung et al. found that at 14–22 weeks, women presenting with a placenta previa were at higher risk of placenta accreta if serum *β*-hCG is above 2.5 MOM (OR 3.9, 95% CI 1.5–9.9) [80]. Thompson et al. examined routine first trimester maternal serum free *β*-hCG measurements comparing between normal pregnancies (*n* = 344), placenta previa (*n* = 155), and PAS (*n* = 17) [58]. Median free *β*-hCG was significantly reduced in the PAS group (Table 1) with a significantly different distribution from controls (*P* = 0.031).

By contrast, in a larger retrospective case-control study, Dreux et al. compiled a database consisting of 69 PAS patients who underwent routine second trimester maternal aneuploidy screening. The control group consisted of 552 serum samples (1 : 8 ratio) matched by maternal age, randomly selected from the routine second trimester maternal serum screening databases [81]. Second trimester free *β*-hCG was significantly higher, 1.50 MoM versus 1 MoM (*P* < 0.0001), in cases of clinically suspected and/or histologically confirmed cases of PAS (*P* < 0.0001) versus the control group. This study, however, was limited by the exclusion of cases that were treated conservatively (34 of 69). Since conservatively managed cases are also a part of the morbidly adherent placenta continuum, the exclusion of these cases was questioned for potentially affecting the study result. Interestingly, Desai et al. did not find any significant difference in second trimester free *β*-hCG values between cases of placenta previa with PAS and nonadherent placenta previa [69].

Based on these findings, combining PAPP-A and free *β*-HCG may have utility. Bueke et al. investigated 88 patients with placenta previa, 19 of which had PAS. Free *β*-HCG and PAPP-A values in the first trimester screening tests were significantly higher in PAS cases compared to control pregnancies [82]. It is likely that with additional larger study populations, a multiparameter test for PAS including sonographic and MRI findings and serum biomarkers could be developed.

### 4.3. Alpha-Fetoprotein (AFP)

Alpha-fetoprotein (AFP) is produced by the yolk sac and the fetal liver during fetal development [82]. It is thought to be the fetal analog of serum albumin, binds to copper, nickel, fatty acids, and bilirubin and exists in monomeric, dimeric, and trimeric forms [84]. Maternal plasma levels peak near the end of the first trimester begin decreasing at that time and then decrease rapidly after birth. Normal adult levels are usually achieved by the age of 8 to 12 months. The function of AFP in adult humans is unknown; however, in rodents, it binds to oestradiol to prevent transplacental transport and prevent the virilisation of female fetuses. As human, AFP does not bind oestrogen; its function in humans is less clear [85].

Unexplained high levels of maternal serum AFP in the second trimester are associated with adverse pregnancy outcomes [86]. This led to further studies into maternal serum AFP in the setting of a nonanomalous fetus, to investigate any association between elevated second trimester maternal serum AFP and PAS, all of which suggest that increased level of AFP are associated with PAS (Table 1) [86]. Interestingly, Zelop et al. showed that although AFP was elevated in approximately 45% of confirmed PAS cases, the negative controls did not have elevated levels suggesting this test may have a high negative predictive value [57].

### 4.4. Cell-Free Fetal DNA (cffDNA)

Lo et al. first described the detection of fetal DNA in maternal plasma and serum in 1997 [87]. A pilot study showed increased cffDNA in cases of placenta previa with the highest levels seen in the two patients with PAS [88]. Samuel et al. investigated whether antenatal levels of cffDNA could predict PAS compared to patients with uncomplicated placenta previa and to women with prior caesarean section delivery (CD) and normal placentation [89]. The mean fraction of cffDNA did not differ significantly by group when controlling for maternal weight, placental weight, number of prior CD, or years from prior CD. The mean gestational age of sampling of the cffDNA was 34 weeks, and given it may also be possible that the abnormality in placental invasion (possibly correlating with increased apoptosis and release of cffDNA) is a process much earlier in gestation, it was suggested that perhaps a significant difference between groups would be seen at an earlier gestation. As a result further studies are required in this area including longitudinal studies from earlier gestations and experiments combining ccfDNA and placental profiling to assess specific markers.

### 4.5. Cell-Free Placental mRNA

Circulating cell-free placental mRNA has emerged as a potential marker because it can be stably isolated and quantified from maternal plasma [90–94]. El Behery et al. investigated whether measuring cell-free placental mRNA in maternal plasma improved the diagnostic accuracy of ultrasound and colour Doppler in detecting placental invasion in patients at risk for PAS [94]. Thirty-five singleton pregnant women (7 with confirmed PAS) of more than 28 weeks of gestation and at risk of PAS underwent ultrasound and colour Doppler assessment. Cell-free placental mRNA in maternal plasma was measured using real-time reverse transcription polymerase chain reaction (rt-PCR) targeting human placental lactogen.

The median MoM value of cell-free placental mRNA was significantly higher in patients with placenta accreta than in those without placenta accreta (6.50 versus 2.60; *P* < 0.001). Moreover, cell-free placental mRNA appeared to correlate with the degree of placental invasion. There were six false-positive ultrasound diagnoses, all of whom had an insignificant rise in cell-free placental mRNA level suggesting that measuring cell-free placental mRNA in maternal plasma may increase the accuracy of ultrasound and colour Doppler in prenatal prediction of PAS.

In 2012, Zhou et al. measured cell-free *β*-HCG mRNA concentrations from maternal plasma samples at 28–30 weeks gestational age to determine their applicability for antenatal diagnosis of PAS (*n* = 12) when compared with patients with placenta previa alone (*n* = 21) and with women with prior CD and normal placentation (*n* = 35) [94]. The plasma mRNA levels of *β*-HCG were found to be higher in the PAS group, whereas no significant difference was detected in the plasma *β*-HCG protein levels.

## 5. Conclusion

In summary, there are a limited number of studies that have contributed to our understanding of the molecular biology of PAS; however, it does bear many similarities to cancer biology. Chronic basal inflammation combined with a failure of normal placental apoptosis appear to partially explain the underlying biology of invasive placentation with associated angiogenesis. Further studies are needed to fully understand these processes.

A number of potential serum biomarkers have been investigated in PAS. They have shown variable reliability and variability of measurement depending on gestational age at sampling. At present, a sensitive serum biomarker for invasive placentation remains elusive [58, 70, 94]. With further supporting data from larger study populations, it may be possible for biomarkers to be combined with sonographic and MRI imaging to screen for PAS antenatally in a model similar to that used for aneuploidy screening. The benefit of this remains unknown until more prospective data are available [95]. Areas for future research may include those biomarkers in use at preclinical/investigational level in the investigation of aneuploidy and disorders of placentation such as preeclampsia. Optimal timing of marker sampling for suspected cases of PAS also warrants further investigation.

### 5.1. Future Work

Our understanding of the underlying molecular biology of PAS is limited. The current evidence supports the theory that PAS occurs due to a failure of a normal decidua to form, which may form an invasive niche similar to the metastatic niche seen in cancer biology. This may result from a deficient endometrium, such as in the presence of a uterine scar, or where there is no normal endometrium to transform into decidua, such as in a tubal ectopic pregnancy [96]. A number of studies have shown that tubal ectopic pregnancy results in immunologically normal and hormonally active trophoblast cells [97, 98]. Therefore, tubal ectopic pregnancies may offer a model to gain more insight into the molecular processes underlying invasive placentation. Furthermore, additional research is needed that compares uterine specimens of PAS to those with a uterine scar and no evidence of PAS. An improved understanding of this biology may allow us to implement novel preventative strategies in the future leading to a reduction in this profoundly morbid condition.

## Figures and Tables

**Table 1:** Summary of published studies examining serum biomarkers for PAS.

Marker	Study	Total cases	Control cases (type)	Year	Median MoM (PAS)	Median MoM (control)	Trimester	PAS histological confirmation	Comment
PAPP-A	Desai et al. [69]	82	66 (previa)	2014	1.68	0.98	First	Yes	Assayed 81–95 days GA
	Thompson et al. [58]	516	344 (normal) 155 (previa)	2015	1.22	1.01	First	Yes	Assayed 11–13 weeks GA
	Lyell et al. [70]	736	699 (previa)	2015	N/A	N/A	First	No	Assayed 10–13 + 6 weeks GA
	Büke et al. [82]	88	69 (previa)	2018	1.20	0.865	First	Yes	N/A
*β*-HCG	Hung et al. [80]	9349	9321 (nonaccreta)	1999	1.7+/−1.1	1.2+/−1.1	Second	2 of 28 (remainder clinical diagnosis)	Assayed 14–22 weeks GA
	Dreux et al. [81]	69	552 (randomly selected routine age-matched maternal serum samples)	2012	1.50	1.00	Second	35 of 69 had surgical treatment—unclear number having histological confirmation	
	Desai et al. [69]	82	66 (previa)	2014	1.00	1.01	Second	Yes	
	Thompson et al. [58]	516	344 (normal) 155 (previa)	2015	0.81	1.04	First	Yes	Assayed 11–13 weeks GA
	Büke et al. [82]	88	69 (previa)	2018	1.42	0.93	First	Yes	N/A
AFP	Zelop et al. [57]	25	14 (previa)	1992	2.3–5.5	Normal range	Second	Yes	Only 45% of PAS had elevated AFP (>2.0 MoM)
	Kupferminc et al. [99]	44	24 (emergency caesarean hyster; no PAS on histopath)	1993	2.7–40.3	Normal range	Second	Yes	Only 45% of PAS had elevated AFP. (≥2.5 MoM)
	Hung et al. [80]	9349	9321 (nonaccreta)	1999	1.7+/−1.0	1.1+/−0.4	Second	2 of 28 (remainder clinical diagnosis)	Assayed 14–22 weeks GA
	Dreux et al. [81]l	69	552 (randomly selected routine age-matched maternal serum samples)	2012	1.23	0.99	Second	35 of 69 had surgical treatment—unclear number having histological confirmation	
	Lyell et al. [70]	736	699 (previa)	2015	N/A	N/A	Second	No	Assayed 15–20 weeks
	Oztas et al. [95]	316	204 (previa) + 61 (PAS managed conservatively)	2016	1.28+/−	0.87+/−0.37	Second	Yes	Assay 16–20 weeks. Cases = PAS requiring hysterectomy

PAPPA: pregnancy-associated plasma protein A; GA: gestational age; AFP: alpha fetal profile; *β*-HCG: human chorionic gonadotropin beta subunit; PAS: placenta accreta syndrome; MoM: multiples of the median.
